# The Impact of Rosemary and Ginger Extracts on Aging and Healthspan in *Drosophila*

**DOI:** 10.14336/AD.2024.1558

**Published:** 2026-02-26

**Authors:** Ricardo Aparicio, Anna M. Salazar, Edward T. Schmid, Armen Khanbabaei, Arun Rajgopal, R. Keith Randolph, David W. Walker

**Affiliations:** ^1^Department of Integrative Biology and Physiology, University of California, Los Angeles, Los Angeles, California 90095, USA.; ^2^Department of Molecular Biology and Biochemistry, Christopher Newport University, Newport News, Virginia 23606, USA.; ^3^Arkansas Biosciences Institute, Arkansas State University, Jonesboro, Arkansas 72467, USA.; ^4^Department of Biological Sciences, Arkansas State University, Jonesboro, Arkansas 72467, USA.; ^5^Amway Innovation and Science, Ada, Michigan 49355, USA.; ^6^Molecular Biology Institute, University of California, Los Angeles, Los Angeles, California 90095, USA.

**Keywords:** Geroscience, Anti-Aging, Botanicals

## Abstract

Aging leads to a decline in physiological functions and increased risk of mortality, yet therapeutic avenues are limited. Dietary phytochemicals provide an attractive approach to counteract age-related health decline. Here, we have examined the impact of feeding extracts of rosemary and ginger, prepared via three different extraction methods, on markers of aging and healthspan in the fruit fly *Drosophila*. We observed that certain, but not all, extracts of ginger produce modest prolongevity effects. Feeding extracts of rosemary, produced via the three different methods, each produced prolongevity effects. We observe that feeding combinations of both rosemary and ginger extracts leads to robust lifespan extension. We find that the prolongevity effects of rosemary and ginger extracts are linked to improved intestinal barrier function in aged flies. Importantly, we show that the anti-aging effects observed are not linked to reduced food intake. Interestingly, we observe several instances where the combination of rosemary plus ginger produces effects which are more pronounced or not seen for either extract alone. In terms of cellular hallmarks of aging, rosemary plus ginger feeding leads to AMPK activation and improved markers of autophagy and proteostasis in aged flies. Furthermore, feeding the combination of rosemary plus ginger feeding improves cognitive function in aged flies. Our results demonstrate that rosemary and ginger extracts can counteract aging and prolong healthspan in flies.

## INTRODUCTION

Advances in science and medicine, along with improved living conditions in developed countries, have dramatically increased human lifespan in the preceding decades. However, as getting older is the major risk factor for debilitating conditions, including cancer, cardiovascular disease and neurodegeneration [1, 2], new societal and biomedical challenges have arisen. Indeed, as aging impairs sensory and motor function, thereby reducing quality of life, there is a growing realization that focusing upon identifying interventions that can prolong healthy, disease-free lifespan (healthspan) should be a priority [2-4]. In recent decades, aging research has been revolutionized by the use of laboratory model organisms, which recapitulate many of the features of human aging [5]. The fruit fly *Drosophila melanogaster* has been a key model in expanding our understanding of the biological mechanisms of aging and age-onset health decline [6]. Studies in multiple model organisms, including *Drosophila* and mammalian models, have shown that intestinal barrier dysfunction is a conserved patho-physiological feature of aging [7]. Critically, loss of intestinal barrier function is coupled to systemic inflammation, organismal health decline and mortality [7-13]. Hence, treatments that can forestall or prevent age-onset intestinal barrier dysfunction offer enormous potential to prolong healthspan. Brain function also declines with age, including changes in cognitive function[14-16], reducing quality of life in the elderly. In addition, aging is the major risk factor for the development of neurodegenerative diseases, including Alzheimer’s disease and Parkinson’s disease, which have increased in prevalence in recent years[17]. Hence, there is a considerable need for interventions that can delay markers of brain aging.

Geroscience research aims to target the cellular drivers of the aging process to prevent the loss of function that accompanies aging and, thereby, prolong healthspan [3, 18]. This approach, therefore, requires a detailed understanding of how molecular and cellular events that occur during aging manifest as age-onset health decline. In recent years, considerable progress has been made in identifying the cellular hallmarks of aging[19], which have been defined by studies showing that they represent therapeutic targets to slow aging. Autophagy, an intracellular lysosomal degradation pathway has emerged as an important modulator of tissue and organismal aging [20]. In this process, cellular materials (autophagic cargo) are sequestered within double-membrane vesicles known as autophagosomes, and delivered to the lysosome for degradation [21]. Critically, disabled autophagy is widely recognized as a primary hallmark of aging [19, 22], indicating that a failure to turnover autophagic cargo is a driver of age-onset health decline. Another key hallmark of aging is a loss of protein homeostasis (proteostasis), leading to the accumulation of misfolded, or ubiquitinated proteins that can form aggregates as intracellular inclusion bodies [19, 23]. Hence, it stands to reason that interventions that can activate autophagy and/or improve proteostasis during aging could provide therapeutic avenues to prolong healthspan. In this regard, AMP-activated protein kinase (AMPK), the principal energy sensor in eukaryotic cells, has been implicated as a therapeutic target to counteract aging and age-onset pathologies [24-26]. AMPK activation promotes energy homeostasis by increasing catabolic pathways while switching off biosynthetic pathways [26]. More specifically, AMPK can stimulate autophagy by reducing Target of Rapamycin (TOR) signaling and, also, via direct phosphorylation of the protein kinase that initiates autophagy, ULK1 [27, 28]. Critically, numerous studies in model organisms have shown that genetic induction of AMPK can extend lifespan [29-33]. Moreover, it has been shown that AMPK-mediated longevity appears to proceed via autophagy activation and improved proteostasis during aging [31]. AMPK activation has been implicated in the health and longevity benefits of both dietary restriction (also referred to as caloric restriction) and exercise [25, 34, 35] and there is enormous interest in identifying additional approaches to activate AMPK to promote healthspan [36-38].

Research on the health-promoting effects of natural products, including botanical extracts, is often motivated from traditional use, or from evidence that increased consumption of plants is associated with better health outcomes [39, 40]. Ginger (*Z*. *officinale* Roscoe) has been reported to exert extensive pharmacological activities, including antioxidant, anti-inflammatory and anti-tumor [41-44]. Moreover, ginger extracts have been reported to improve outcomes in models of Alzheimer’s disease [45], Parkinson’s disease [46] and diabetes [42]. Ginger is composed of two major active components, 6-gingerol and 6-shogaol, that contribute to many of the biological activities of ginger [47, 48]. Rosemary *(Salvia rosmarinus;* formerly referred as *Rosmarinus officinalis Linn*) has also been shown to exert various beneficial pharmacological effects, including anti-inflammatory, antioxidant, and anti-apoptotic [49-51]. The main relevant constituents are composed of polyphenolics, including carnosic acid, carnosol, rosemarinic acid and ursolic acid [49, 52]. Several studies have reported that rosemary can exert protective effects on both brain health and gut health[49, 50, 53, 54]. In addition, it has been reported that feeding ginger extract, 6-gingerol or 6-shogaol can prolong lifespan in flies [55] and worms [56-58]. It has also been reported that rosemary extract can protect against the lifespan-shortening effects of a high fat diet in flies [59].

However, the ability of rosemary extracts to prolong fly lifespan under normal dietary conditions has not been reported. Indeed, numerous questions remain regarding the potential anti-aging effects of rosemary and ginger extracts, including whether any prolongevity effects reported are linked to reduced food intake, which would be a confounding factor in any longevity study. At the same time, the interplay between rosemary and/or ginger and the cellular hallmarks of aging remains poorly understood. Moreover, further work is required to establish a definitive role for either ginger extract or rosemary extract in modulating healthspan, as opposed to lifespan.

One promising area of research is testing the combinatorial effects of anti-aging interventions [60]. In this study, we set out to determine whether rosemary and/or ginger extract feeding can counteract cellular hallmarks of aging, delay age-onset pathophysiology and/or prolong healthspan. We examined three different types of rosemary and ginger extracts; prepared via chloroform extraction, ethanol extraction and binary solvent (ethanol-water) extraction. We find that feeding ginger extract prepared via absolute ethanol extraction can modestly prolong lifespan and delay the onset of intestinal barrier dysfunction. We observe that feeding rosemary extract, prepared via all three different extraction methods, extends lifespan and counteracts age-onset intestinal barrier dysfunction. Critically, we exclude the possibility that the prolongevity effects of rosemary or ginger extracts are due to decreased food intake. Focusing on ethanol and the ethanol-water extracts of both Rosemary and Ginger, we observe that feeding flies rosemary extract plus ginger extract leads to AMPK activation and improved proteostasis, neither of which was observed for rosemary or ginger extracts alone. We also observe that feeding rosemary extract and ginger extract leads to improved markers of autophagy in aged flies. Finally, we observe that feeding flies rosemary extract leads to improved cognitive performance during aging. Interestingly, the effect on cognition in aged flies of feeding both rosemary extract and ginger extract is more pronounced than feeding rosemary extract alone.

## MATERIAL AND METHODS

### Botanical Extracts

#### Chloroform extraction

Rosemary (*Salvia rosmarinus*) leaves and sage (*Salvia officinalis*) aerial parts were sourced from Amway owned farm in Jalisco, Mexico. Ginger roots (*Zingiber officinale*), boldo (*Vernonia condenseta*) aerial and carqueja (*Baccharis crispa*) aerial parts were sourced from Amway owned farm in Ubajara, Brazil. The plants were first harvested and dried at room temperature. The dried leaves, roots or aerial parts were then milled. To this milled powder 300 mL 100% Reagent Grade Chloroform (1:3 w/v) was added. It was covered and stirred overnight (16-18 hours). The next day the sample was sonicated for 1 hour. The sample was filtered twice through a Watman GF/A filter paper into a round bottom flask. The volume of the extract was reduced using a rotary evaporator at 40°C to about10 ml. The liquid was transferred to 30 mL vial and dried using nitrogen gas until all of the chloroform was removed. The vial was then placed in a desiccator for several days to complete dryness and was stored at -80°C until further use.

#### Absolute Ethanol extraction

Rosemary (*Salvia rosmarinus*) leaves were sourced from Amway owned farm in Jalisco, Mexico or Ginger roots (*Zingiber officinale*) were sourced from Amway owned farm in Ubajara, Brazil were first harvested and dried at room temperature. The dried leaves or roots were milled. To this milled powder, 300 mL 100% Reagent Grade Ethanol (1:6 w/v) was added. It was covered and stirred overnight (16-18 hours). The next day the sample was sonicated for 1 hour. The sample was filtered twice through a Watman GF/A filter paper into a round bottom flask. The volume of the extract was reduced using a rotary evaporator at 40-45°C to about 10 ml. The liquid was transferred to 30 ml vial and dried using nitrogen gas until all of the ethanol was removed. The vial was then placed in a desiccator for several days to complete dryness and stored at -80°C till further use.

#### Water-Ethanol extraction

Rosemary (*Salvia rosmarinus*) leaves sourced from Amway owned farm in Jalisco, Mexico were first harvested and dried at room temperature. The leaves were then milled to a fine powder. Then a two stage ethanolic extraction was performed. Stage 1 extraction was carried out with 83% ethanol (1:6.5 w/w) at 60°C for 120 mins with constant stirring. The extract was filtered through 80 mesh bag, and the residue was extracted a second time with 85% ethanol (1:5.5 w/w) at the same temperature for 60 mins. The extracts were then filtered again through 80 mesh bags. Extracts from step 1 and step 2 were then combined and concentrated with ethanol removed by using a rotatory evaporator. The extract was then freeze-dried and stored at -80°C until further use.

The ginger extract was obtained from botanical extract supplier PLT health solutions (Morristown NJ). Ginger (*Zingiber officinale*) roots sourced from farms in India, China and Nigeria, were collected and dried at room temperature. The roots were milled to a fine powder and extracted with 80% ethanol (1:10 w/w) for 180 mins at 60 °C and then filtered with 80 mesh bags. The extract was then concentrated, and ethanol removed via rotatory evaporator. The extract was then freeze dried and stored at -80°C until further use.

For both the ethanol and the ethanol-water extracts, the rosemary extract used in the study was standardized to not less than 1% rosmarinic acid. The rosmarinic acid assay was performed via a UHPLC method that uses a C18 column, UV detection, and a ternary mobile phase gradient consisting of acid-modified water, methanol, and acetonitrile. The ginger extract used in the study was standardized to not less than 5% total pungents (6, 8, & 10-gingerol and 6, 8, & 10-shogaol). The pungents assay was performed on an HPLC system using a C8 column, UV detection, and a binary mobile phase gradient consisting of acid-modified water and acetonitrile.

### Fly stocks, husbandry and lifespan assay

The *w^dahomey^* (*w^dah^*) wild type strain was used in this work. Flies were reared in vials containing cornmeal medium (1% agar, 3% yeast, 1.9% sucrose, 3.8% dextrose, 9.1% cornmeal, 1.1% acid mix (41.8% Propionic acid plus 4.15% Phosphoric acid in vol/vol), and 1.5% methylparaben (10% methylparaben in ethanol in wt/vol), all concentrations given in wt/vol). Flies were collected under light nitrogen-induced anesthesia and housed at a density of 30 female flies per vial. All flies were kept in a humidified, temperature-controlled incubator with 12 h on/off light cycle at 25°C. All extracts independently of the method used for their extraction were diluted in ethanol and added to food at the indicated concentration in each figure, ethanol was used as a control.

### CApillary FEeding Assay (CAFE Assay)

Food intake was analyzed at day 10 using the CApillary FEeding (CAFE) assay as described [61] with modifications. Flies were fed from day 3 onwards with or without the specific extract added to the food. 80 flies per condition were tested, 10 flies were placed in vials with wet tissue paper at the bottom and a capillary food source [5% sucrose, 5% yeast extract, 2.5% FD&C Blue No. 1 (SPS Alfachem), 2.5mg/ml extract or ethanol]. Feeding was monitored for 10 hours, during light time and 30 min. after lights go off, feeding amount was recorded every 1-2 hours and the capillaries were replaced frequently.

### Consumption-excretion assay

Consumption-excretion assays were performed as previously described in [62] with modifications. Flies were fed from day 3 onwards until the day of the analysis with or without extracts. At the day of the analysis flies were transferred to new empty vials (six vials with 10 flies each) and fed from feeder caps containing standard medium with 2.5% wt/vol F&D blue dye no 1 and extracts or ethanol for 20 h at 25°C. Feeder caps were discarded at the conclusion of feeding. For checking internal (consumed food) dye, flies were homogenized in 500 μl of double-distilled H_2_O (ddH_2_O) and pellet debris was removed by centrifugation. The dye excreted by the flies on the walls of the vials was collected by the addition of 1 ml of ddH_2_O to each vial and vortexing. Samples were quantified using an Epoch BioTek microplate reader and compared to a serially diluted standard.

### Intestinal barrier dysfunction (Smurf) assay

Intestinal integrity assays were performed as previously described [12]. To conduct the ‘Smurf assay’, flies were then transferred to new vials containing standard medium with 2.5% wt/vol F&D blue dye # 1 (SPS Alfachem) for 16 hours. The number of flies per vial with dye coloration outside the gut (Smurf flies) were then tallied and quantified.

### Olfactory Training

Aversion training was performed as described in [63] using a system from MazeEngineers (Conduct Science). Briefly, flies were exposed to a neutral odor (3-octanol) by air pump in a training chamber for one minute under low red-light conditions. They were exposed to the odor in a series of twelve 60-V shocks for 1.25 seconds followed by rest for 3.75 seconds for a total of one minute. Flies recovered for one hour before being placed in a T-maze with trained odor on one side and a second neutral odor (4-methylcyclohexanol) on the other side of the maze. After two minutes of exploration under red-light conditions, flies in either chamber of the maze were scored. The performance index was calculated by dividing the number of flies avoiding the training odor by the number of participants.

### Genomic DNA isolation

Genomic DNA was extracted using the PowerSoil DNA isolation kit (MoBio). All flies were surface sterilized as previously described [8] prior to sample preparation. To ensure consistent homogenization, whole fly (10 flies) samples were pre-homogenized in 150-µL of solution from the PowerSoil bead tube using a motor pestle. This homogenate was then returned to the bead tube, and the manufacturers protocol was followed.

### qPCR

qPCR was performed with PowerUP SYBR Green Master Mix (Ref#A25777, Applied Biosystems) on a BioRad Real Time PCR system. Cycling conditions were as follows: 95°C for 10 minutes; 95°C for 15s then 60°C for 60s, cycled 45 times, and equalized amplicons of Actin5C were used as a reference to normalize. The following primers sequence were used: Act5C_F: TTGTCTGGG CAAGAGGATCAG; Act5C_R: ACCACTCGCACTTG CACTTTC; 16S_F: AGAGT TTGATCCTGGCTCAG; 16_R: CTGCTGCCTYCCGTA.

### Immunostaining and image analysis

Flies were fixed in 3.7% formaldehyde in PBS for 20 minutes. After fixation hemi-thoraxes were dissected and fixed again for 5 min. Samples were then rinsed 3 times for 10 min. with 0.2% Triton X-100 in PBS (PBST) and blocked in 3% BSA in PBST (PBST-BSA) for 1 hour. Primary antibodies were diluted in PBST-BSA and incubated overnight at 4°C. Primary antibodies used were: mouse-anti-FK2 1:250 (BML-PW8810-0500, ENZO); rabbit-anti-dP62 1:250. Hemi-thoraxes were then rinsed 3 times in PBST for 10 min. and incubated with the secondary antibodies and/or stains at room temperature for 3 hours. Secondary antibodies and dyes used were: anti-mouse AlexaFluor-488 1:500 (Invitrogen); anti-rabbit AlexaFluor-647 1:500 (Invitrogen); phalloidin AlexaFlour-568 1:250 (Invitrogen). Finally, samples were rinsed 3 times with PBST for 10 min. and mounted in Vectashield Mounting Medium (Vector Lab). Images were taken using Zeiss LSM780 or LSM 880 Airyscan confocal microscope and analyzed using the ImageJ software to measure ubiquitin and dP62 aggregates sizes.

### Western blot

Thoraxes (5 thoraxes per sample) were homogenized in Lysis Buffer (PBS 1X, Protease Inhibitors 1X (5892791001, Sigma-Aldrich), NuPAGE LDS Sample Buffer 1X (NP0001, Thermo Fisher Scientific), and DTT (Dithiothreitol) 0.05M). Samples from whole flies were separated by SDS-PAGE gels and proteins were transferred to Nitrocellulose membranes. Membranes were probed with antisera against: anti-a-actin peroxidase conjugated 1:15000 (A3854, Sigma), rabbit anti-GABARAP (ab109364, Abcam) 1:1000, rabbit anti-p-AMPK (2535S cell Signaling) 1:1000, and mouse-anti-ubiquitin (P4D1) (3936, Cell Signaling) 1:1000. Anti-Rabbit or anti-Mouse Horseradish peroxidase conjugated antibodies were used for detection at 1:10000 dilution. Amersham ECL Prime Western Blotting Detection Reagent (GE life sciences) was used to visualize the presence of horseradish peroxidase, and the chemiluminescent signal was recorded using Syngene Pxi Western Blot Imager. Image analysis was done using ImageJ.

### Quantification and Statistical Analysis

GraphPad Prism 10 was used to perform the statistical analysis and graphical display of the data. Significance is expressed as p values as determined by two-tailed. Gaussian distribution with parametric distribution was used when samples reach the distribution criteria, or non-parametric distribution was used when samples did not reach the criteria for Gaussian distribution. For comparisons of two groups, an unpaired t test was used. For comparisons of more than two groups, one-way ANOVA with Šídák correction was performed, or Kruskal-Wallis tests with Dunn’s multiple comparisons post hoc tests was used when data do not reach the criteria for one-Way ANOVA analysis. Scatter plots with bar depict mean ± SEM. The difference between two groups was defined as statistically significant for the following p values: * < 0.05, ** < 0.01, *** < 0.001, and ****<0.0001. The number (n) of biological samples used in each experiment and what n represents can be found in each figure legend.

Log-rank (Mantel-Cox) test was used for survival curves comparison. Average median survival is the time point at which the probability of survival equals to 50%. The difference between survival curves was defined as statistically significant as follow: * < 0.05, ** < 0.01, *** < 0.001, and ****<0.0001.

**Table 1:** Impact of chloroform botanical extracts on *Drosophila* lifespan.

	Concentration (mg/ml)	Median lifespan	p-value versus control	% median lifesapn increase	n
**Ginger**	0	47			326
	**0.5**	50	0.1642	6.4	322
	**2.5**	52	0.209	10.6	332
**Rosemary**	0	42			260
	**0.1**	44	0.0009	4.8	286
	**0.5**	44	0.0478	4.8	269
	**2.5**	44	0.0006	4.8	296
**Rosemary/ginger**	0	42			279
	**0.1**	47	0.0069	11.9	281
	**0.5**	50	0.0032	19	289
	**1.25**	50	0.0664	19	291
**Boldo**	0	44			241
	**0.1**	42	0.8641	-4.5	214
	**0.5**	42	0.7177	-4.5	219
	**2.5**	39	0.0003	-11.4	174
**Sage**	0	46			245
	**0.5**	49	0.9334	6.5	241
	**2.5**	46	0.4386	0	228
**Carqueja**	0	42			295
	**0.1**	42	0.9419	0	277
	**0.5**	42	0.5365	0	261
	**2.5**	44	0.047	4.8	255

## RESULTS

### Chloroform extracts of rosemary and ginger can prolong *Drosophila* lifespan

**Figure 1.**
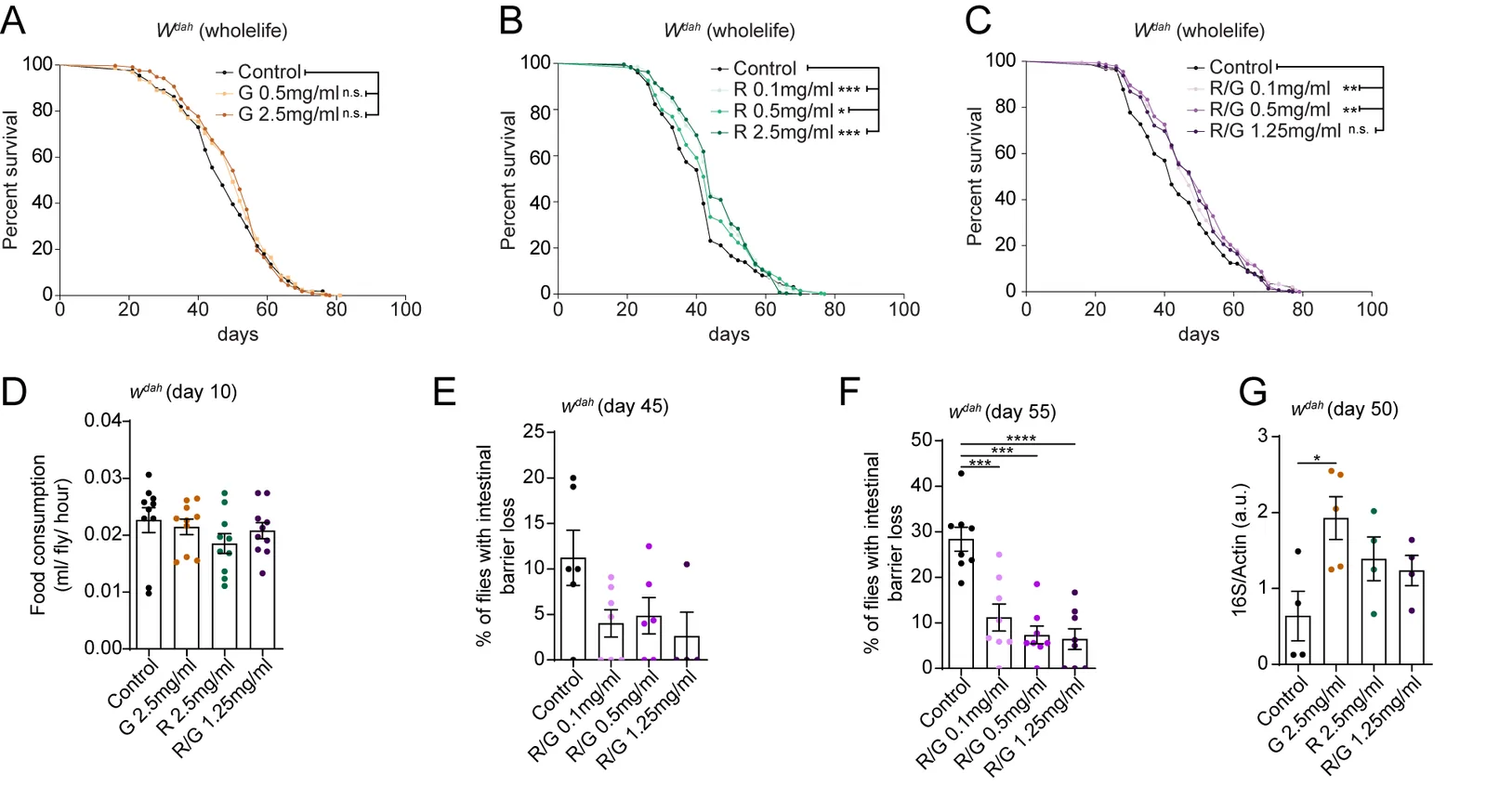
**Chloroform extraction of Rosemary and ginger prolongs *Drosophila* lifespan and healthspan**. (**A-C**) Survival curves of *w^dah^* female flies fed from day 3 onwards with different botanical extracts at different concentrations. (**A**) ginger, (B) rosemary, (C) rosemary plus ginger, log-rank test. For statistical analysis see Table 1. (**D**) CApillary Feeding Assay (CAFE) of 10 days old *w^dah^* females fed form 3 day onwards with ethanol (control), ginger, rosemary, and rosemary/ginger. n = 10 replicates of 10 flies per replicate; one-way ANOVA/Šídák multiple-comparisons test. (E and F) Intestinal barrier loss in *w^dah^* female flies at day 45 (E) and 55 (F) fed from day 3 onwards with ethanol (control), and rosemary/ginger. For day 45 control n = 6, R/G 0.1 mg/ml n = 7, R/G 0.5 mg/ml n = 6 and R/G 1.25 mg/ml n= 4 vials with 30 flies per vial. For day 55 control n = 8, R/G 0.1 mg/ml n = 8, R/G 0.5 mg/ml n=8, and R/G 1/25 mg/ml n = 8 with 30 flies per vial; ***p0.001; and ****p0.0001; Kruskall-Wallis/Dunn’s multiple-comparisons test. (**G**) Bacterial levels assayed by taxon-specific qPCR of the 16S ribosomal gene in 50-day-old surface-sterilized *w^dah^* female flies fed from day 3 onwards with ethanol (control), ginger, rosemary, and rosemary/ginger. n= 4 replicates of 5 whole flies for Control, Rosemary and R/G and 5 for Ginger. *p0.05; Kruskall-Wallis/Dunn’s multiple-comparisons test. G = Ginger, R = Rosemary.

First, we examined the impact of several botanical extracts produced by chloroform extraction; rosemary, boldo, carqueja, sage, and ginger on female fly lifespan. To do so, we fed different concentrations of each botanical extract to the wild type strain *w^dahomey^* (*w^dah^*). We failed to observe any major positive effects upon feeding boldo, carqueja, or sage extracts at any of the concentrations tested (Supp Fig. 1A-C; Table 1). However, feeding *w^dah^* flies with extracts of ginger produced a modest increase in median lifespan that was not statistically significant (Fig. 1A; Table 1). However, feeding rosemary extract lead to a ~5% increase in median lifespan at several different concentrations (Fig. 1B; Table 1). Interestingly, feeding a combination of rosemary plus ginger (0.5mg/ml) produced a robust (19%) increase in median lifespan (Fig. 1C; Table 1). As alterations in feeding behavior could be a confounding variable in lifespan studies [64], we examined whether feeding these botanical extracts could affect food intake under these conditions. To do so, we used the CApillary FEeding assay (CAFE) to analyze feeding behavior in control flies and those fed with rosemary extract and/or ginger extract [61].

Importantly, we failed to detect any alterations in feeding behavior upon feeding rosemary, ginger or rosemary plus ginger extracts (Fig. 1D). Next, we set out to determine whether the lifespan-promoting effect of rosemary and ginger extracts were linked to improved intestinal barrier function during aging. In *Drosophila*, intestinal barrier integrity can be monitored by the ‘Smurf assay’, which involves the detection of non-absorbable dyes, fed to flies, outside of the digestive tract [12, 65]. We assayed intestinal barrier integrity in flies fed rosemary extract plus ginger extract at days 45 and 55. At day 45, there was a non-significant trend toward improved intestinal barrier function in flies fed extracts (Fig. 1E). Importantly, we observed a significant decline in the number of flies with intestinal barrier dysfunction at day 55 in all the extract-fed conditions tested in comparison with control flies (Fig. 1F). It is known that the gut microbiota contributes to cellular and physiological changes in the aging intestine and, in some cases, age-related shifts in microbiota dynamics can drive health decline in aged animals [66-68]. Moreover, in flies, it has been shown that reducing the microbial load in the gut can delay the onset of intestinal barrier dysfunction [8]. Hence, to explore the impact on commensal homeostasis during aging, we utilized qPCR with universal primers to the bacterial 16S ribosomal gene to characterize alterations in microbiota dynamics in response to extract feeding. Feeding rosemary extract or rosemary plus ginger extracts for 50 days does not alter the bacterial load, while feeding ginger extract increases the bacterial load (Fig. 1G). Hence, we conclude that the beneficial impact of rosemary and ginger extracts on intestinal barrier function during aging are not linked to reduced intestinal bacterial load. These data demonstrate that chloroform extraction of the botanical extracts rosemary and ginger prolongs *Drosophila* lifespan and delays age-onset pathology, without reducing food intake or decreasing microbial load.

**Figure 2.**
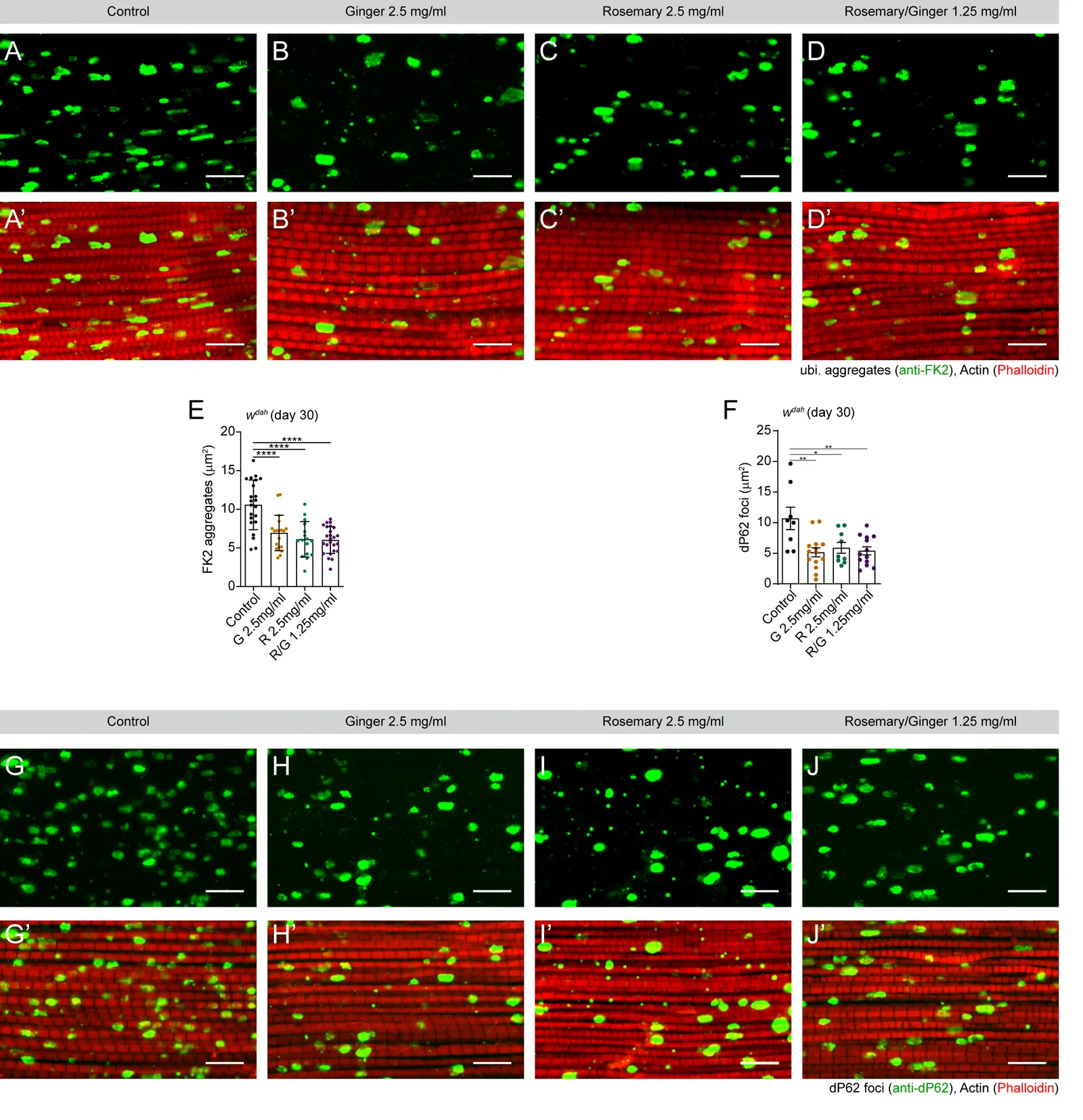
**Chloroform extraction of Rosemary and ginger improves proteostasis**. (A-D’) Immunostaining of indirect flight muscles from day 30 of *w^dah^* female flies fed with ethanol (control), ginger, rosemary, and rosemary/ginger showing polyubiquitinated aggregates (A-D and A’-D’) (green channel, anti-FK2); and muscles (A’-D’) (red channel stained with phalloidin/F-Actin). Scale bar is 10 μm. (**E**) Quantification of polyubiquitin aggregates in muscle as shown in (A-D). Control n = 22 hemi-thoraxes, Ginger n = 19 hemi-thoraxes, Rosemary n = 16 hemi-thoraxes, and R/G n = 25 hemi-thoraxes; ****p 0.0001; one-way ANOVA/Šídák multiple comparisons test. (**F**) Quantification of dP62 aggregates in muscle as shown in (G-J). Control n = 8 hemi-thoraxes, Ginger n = 14 hemi-thoraxes, Rosemary n = 9 hemi-thoraxes, and R/G n = 13 hemi-thoraxes; *p ≤ 0.05; **p ≤ 0.01; one-way ANOVA/Šídák multiple comparisons test. (G-J’) Immunostaining of indirect flight muscles from day 30 of *w^dah^* female flies fed with ethanol (control), ginger, rosemary, and rosemary/ginger showing dP62 aggregates (G-J and G’-J’) (green channel, anti-dP62); and muscles (G’-J’) (red channel stained with phalloidin/F-Actin). Scale bar is 10 μm. G = Ginger, R = Rosemary.

### Chloroform extraction of rosemary and ginger improves proteostasis in aged muscle

Next, we set out to determine whether the prolongevity and anti-aging effects of feeding rosemary and ginger extracts are linked to improved cellular markers of aging. A decline in proteostasis is a major cellular hallmark of aging and numerous age-onset diseases [19, 69]. Hence, we analyzed the accumulation of protein aggregates in *Drosophila* indirect flight muscles. As previously shown, *Drosophila* flight muscles accumulate aggregates of ubiquinated proteins that can be visualized by immunofluorescence (IF) microscopy during aging [31, 70-74]. We fed *w^dah^* flies with rosemary and ginger extracts from day 3 post-eclosion onwards and analyzed the accumulation of protein aggregates on day 30. As shown in Fig. 2A-D (Fig. 2A-D’, and quantification in Fig. 2E), feeding rosemary and ginger extracts to *w^dah^* flies reduces the formation of protein aggregates in the flight muscles of aged flies. Ubiquitinated proteins can be targeted for autophagic degradation by the adaptor protein p62/SQSTM1, with an accumulation of p62 indicating reduced autophagy-mediated turnover [74, 75]. Here, we analyzed the IF accumulation of dP62 in flies fed with rosemary and ginger extracts. Upon 30 days of rosemary extract, ginger extract, and rosemary plus ginger extract feeding, we see a reduction in dP62 foci (Fig. 2G-J and 2G’-J’, and quantification in 2F). These data indicate that rosemary and ginger extracts improve proteostasis during muscle aging.

**Figure 3.**
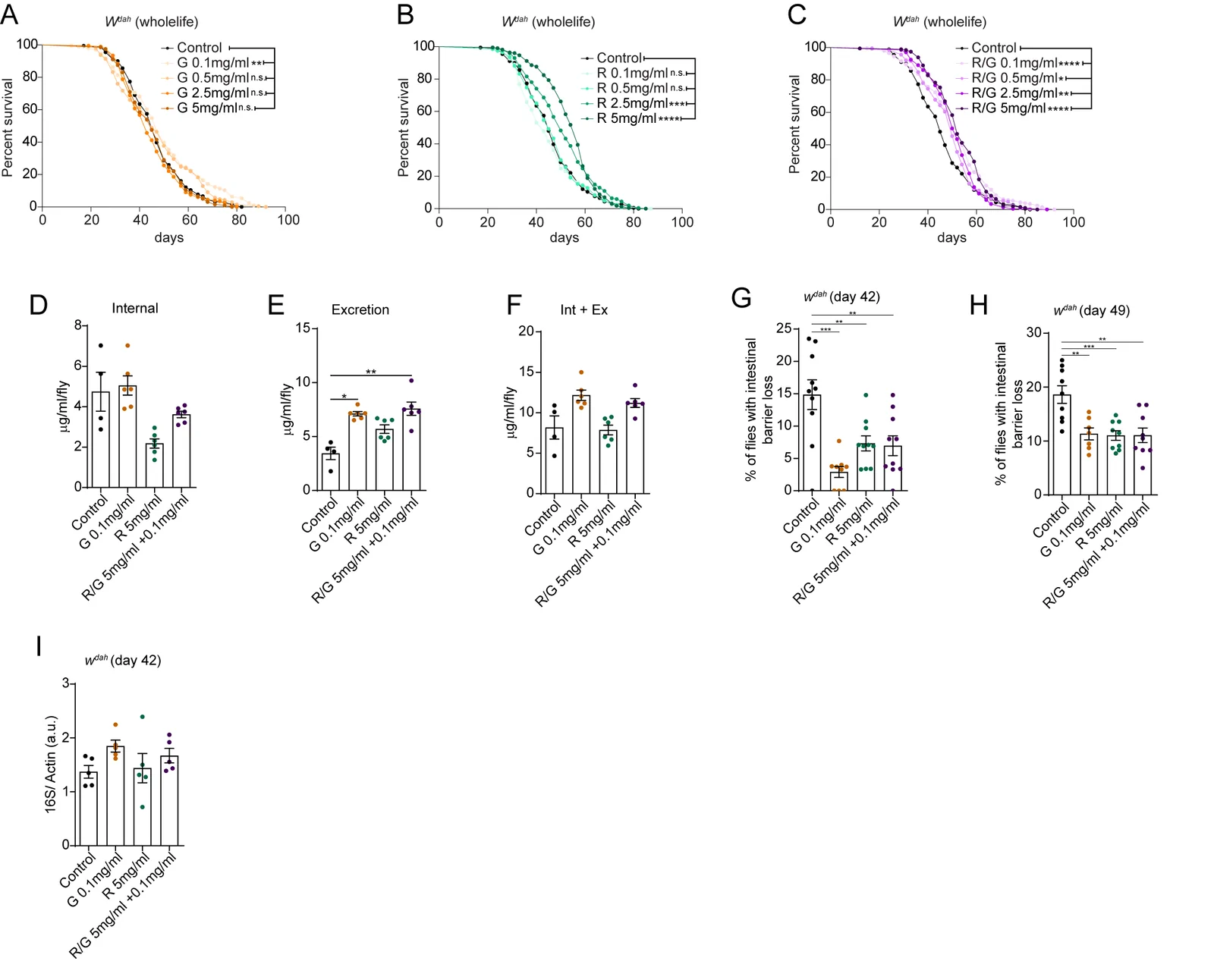
**Effects of absolute ethanol extraction of rosemary and ginger on *Drosophila* lifespan and healthspan**. (**A-C**) Survival curves of *w^dah^* female flies fed from day 3 onwards with different botanical extracts at different concentrations. (**A**) ginger, (B) rosemary, and (C) rosemary/ginger. log-rank test. For statistical analysis see Table 2. (**D-F**) Spectrophotometry analysis of consumed food at day 7 of *w^dah^* female flies fed with ethanol (control), ginger, rosemary, and rosemary/ginger from day 3 onwards. (**D**) internal food, (E) excreted food and (F) internal + excreted (Int + Ex). Control n = 4, Ginger n = 5, Rosemary n = 6, and R/G n = 6 replicates of 10 flies per replicate. *p ≤ 0.05; **p ≤ 0.01; ***p≤0.001; Kruskall-Wallis/Dunn’s multiple-comparisons test. (**G-H**) Intestinal barrier loss in *w^dah^* female flies at day 42 (G) and 49 (H) fed from day 3 post-eclosion onwards with ethanol (control), ginger, rosemary, and rosemary plus ginger. For day 42; Control n = 10, Ginger n = 10, Rosemary n = 9, and R/G n = 10 vials with 30 flies per vial. For day 49; Control n = 9, Ginger n = 9, Rosemary n= 7, and R/G n = 9 vials with 30 flies per vial.; **p ≤ 0.01; ***p≤0.001; one-way ANOVA/Šídák multiple-comparisons test. (**I**) Bacterial levels assayed by taxon-specific qPCR of the 16S ribosomal gene in 42-day-old surface-sterilized *w^dah^* female flies fed from day 3 onwards with ethanol (control), ginger, rosemary, and rosemary/ginger. n = 5 replicates with 5 flies per replicate. Kruskall-Wallis/Dunn’s multiple-comparisons test G = Ginger, R = Rosemary.

### Absolute ethanol extraction of rosemary and ginger extends *Drosophila* lifespan

To determine the generality of these findings with regards to different extraction methods, we examined extracts of rosemary and ginger prepared using absolute ethanol. Feeding low concentration (0.1mg/ml) of ginger to *w^dah^* flies produced a modest increase in median lifespan (Fig. 3A; Table 2). The median lifespan of *w^dah^* flies fed with 2.5mg/ml and 5mg/ml of rosemary significantly extended median lifespan (Fig. 3B; Table 2). Furthermore, feeding multiple concentrations of rosemary plus ginger significantly extended median lifespan (Fig. 3C; Table 2). Interestingly, feeding a low concentration (0.1mg/ml) combination of rosemary plus ginger was able to prolong median lifespan by ~16%, when 0.1mg/ml of rosemary alone or ginger alone either did not extend lifespan or produced a modest ~4% effect (Fig. 3B and C; Table 2). Since rosemary 5mg/ml has the maximum median lifespan extension and ginger 0.1mg/ml prolongs *Drosophila* maximum lifespan this paradigm was used in the following assays. To further explore the impact of rosemary and ginger extracts on feeding behavior, we utilized an assay designed to measure food consumption on solid food [62]. Using this Consumption-Excretion (Con-Ex) feeding assay with a food dye, we examined both food within (internal) and excreted by flies. In this assay, the sum of the dye found inside flies plus the dye excreted from flies reflects overall food intake. Flies fed with rosemary, ginger, and with rosemary plus ginger did not show an overall increase in food intake (Fig. 3F). Interestingly, flies fed with ginger and rosemary plus ginger extracts present higher levels of excreted dye (Fig. 3E). One interpretation of these findings is that rosemary and ginger extracts lead to reduced gut transit time.

We also set out to confirm that ethanol extracts of rosemary and ginger counteract intestinal barrier dysfunction during aging. Indeed, we observed a reduction in the number of flies with intestinal barrier dysfunction at days 42 and 49 when fed with rosemary, ginger, and rosemary plus ginger (Fig. 3G and H). Finally, as was performed for chloroform extracts, we quantified the internal bacterial load by qPCR analysis using universal primers to the bacterial 16S ribosomal gene. Upon 42 days of feeding ethanol extracts of rosemary and ginger, we do not observe any significant changes in internal bacterial load (Fig. 3I). These data demonstrate that feeding flies ethanol extracts of rosemary and ginger can also delay age-onset pathology and prolong healthy lifespan, without reducing food intake or intestinal bacterial load.

**Table 2:** Absolute ethanol extracts of Rosemary and Ginger extend *Drosophila* lifespan.

	Concentration (mg/ml)	Median lifespan	p-value versus control	% median lifespan increase	n (flies)
**Ginger**	0	45			181
	**0.1**	47	0.0032	4.4	204
	**0.5**	45	0.1284	0	209
	**2.5**	43	0.1578	-4.4	199
	**5**	45	0.4324	0	200
**Rosemary**	0	45			181
	**0.1**	43	0.5947	-4.4	290
	**0.5**	45	0.9571	0	203
	**2.5**	50	0.0004	11.1	201
	**5**	57	<0.0001	26.7	228
**Rosemary/ginger**	0	45			181
	**0.1**	52	<0.0001	15.6	203
	**0.5**	50	0.0319	11.1	205
	**2.5**	51	0.007	13.3	202
	**5**	52	<0.0001	15.6	199

### Absolute ethanol extraction of rosemary and ginger improves proteostasis and promotes autophagy

**Figure 4.**
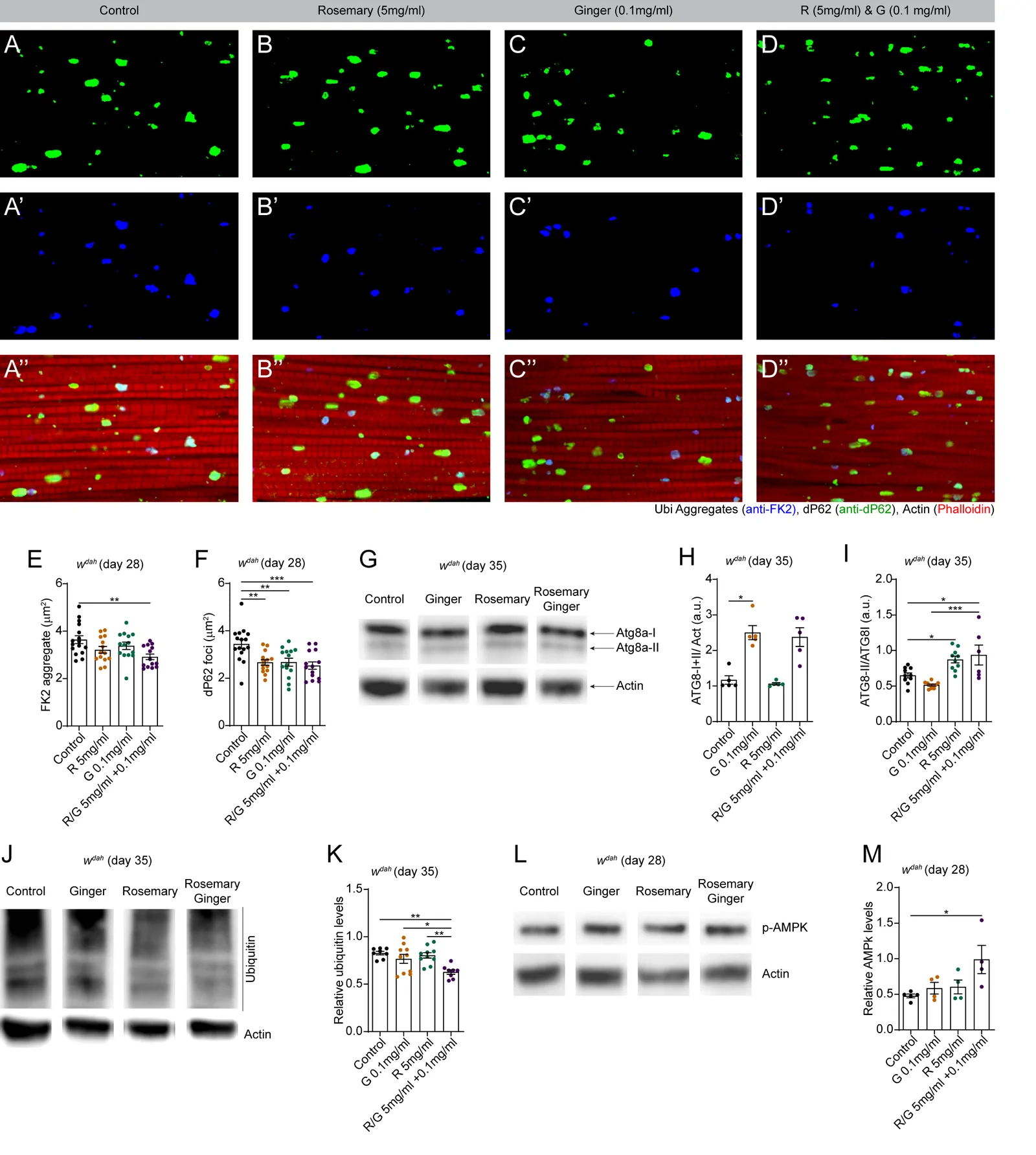
**Absolute ethanol extraction of rosemary and ginger improves proteostasis and activates autophagy and AMPK**. (A-D’’) Immunostaining of indirect flight muscles from day 28 of *w^dah^* female flies fed with ethanol (control), ginger, rosemary, and rosemary/ginger showing polyubiquitinated aggregates (A-D and A’’-D’’) (green channel, anti-FK2); (A’-D’ and A’’-D’’) dP62 aggregates (blue channel, anti-dP62); (A’’-D’’) and muscles (red channel stained with phalloidin/F-Actin). Scale bar is 10 μm. (**E**) Quantification of polyubiquitin aggregates in muscle as shown in (A-D). Control n = 16 hemi-thoraxes, Ginger n = 14 hemi-thoraxes, Rosemary n =14 hemi-thoraxes, and R/G n = 14 hemi-thoraxes; **p 0.01; one-way ANOVA/Šídák multiple comparisons test. (**F**) Quantification of dP62 aggregates in muscle as shown in (A’-D’ and A’’-D’’). Control n = 16 hemi-thoraxes, Ginger n = 14 hemi-thoraxes, Rosemary n =14 hemi-thoraxes, and R/G n = 14 hemi-thoraxes; **p 0.01; ***p 0.0001; one-way ANOVA/Šídák multiple comparisons test. (**G**) Western blot detection of ATG8a levels from day 35 of *w^dah^* females fed with ethanol (control), ginger, rosemary, and rosemary/ginger from day 3 onwards. (**H**) Densitometry analysis of total ATG8a (ATG8a-I + ATG8a-II) levels as shown in (G). Control n = 5, Ginger n= 5, Rosemary n = 5, and R/G n = 5 replicates with 5 thoraxes per replicate; ***p 0.0001; one-way ANOVA/Šídák multiple comparisons test. (**I**) Densitometry analysis of lipidated ATG8a (ATG8a-II) levels as shown in (G). Control n = 10, Ginger n= 10, Rosemary n = 10, and R/G n = 6 replicates with 5 thoraxes per replicate; *p 0.05; one-way ANOVA/Šídák multiple comparisons test. (**J**) Western blot detection of total ubiquitin-conjugated proteins from day 35 *w^dah^* females fed with ethanol (control), ginger, rosemary, and rosemary/ginger from day 3 onwards. (**K**) Densitometry analysis of total ubiquitin-conjugated proteins levels as shown in (J). Control n = 8, Ginger n= 10, Rosemary n = 10, and R/G n = 8 replicates with 5 thoraxes per replicate; **p 0.01; one-way ANOVA/Šídák multiple comparisons test. (**L**) Western blot detection of phosphorylated AMPK (p-AMPK) protein from day 28 *w^dah^* females fed with ethanol (control), ginger, rosemary, and rosemary/ginger from day 3 onwards. (**M**) Densitometry analysis of p-AMPK levels as shown in (L). Control n = 4, Ginger n= 5, Rosemary n = 5, and R/G n = 4 replicates with 5 thoraxes per replicate; *p 0.05; Kruskall-Wallis/Dunn’s multiple-comparisons test. G = Ginger, R = Rosemary.

Next, we set out to determine whether ethanol extracts of rosemary and ginger also improve proteostasis during aging. To do so, we analyzed, by IF, the levels of ubiquitinated proteins and dP62 in aged muscles from *w^dah^* flies fed with ethanol extracts of rosemary, ginger, and rosemary plus ginger from day 3 until the day of the analysis. As shown in Figure 4, individually feeding rosemary and ginger extracts to *w^dah^* flies did not decrease the size of protein aggregates in aged flies. However, feeding rosemary plus ginger to *w^dah^* flies decreases the size of protein aggregates in aged muscles (Fig. 4A-D or A’’-D’’, and quantification in 4E). Moreover, feeding rosemary, ginger, and rosemary plus ginger to flies reduces the accumulation of the autophagy receptor dP62 (Fig. 4A’-D’ or 4A’’-D’’, and quantification in 4F). In a complementary approach, we used western blotting analysis to examine the levels of insoluble ubiquitinated proteins in thoraxes. Consistent with the IF data, we observed that feeding rosemary plus ginger extracts leads to reduced levels of insoluble ubiquitinated proteins during aging (Fig. 4J, and quantification in 4K). dP62 is an autophagy receptor that possesses a C-terminal ubiquitin binding domain and a short ATG8/LC3 interacting region [21]. LC3/ATG8 can be conjugated to phosphatidyl-ethanolamine (PE) with the non-lipidated and lipidated forms referred to as ATG8/LC3-I and ATG8/LC3-II, respectively [75]. Increased levels of PE-modified Atg8/LC3 (ATG8/LC3-II) can reflect the induction of autophagic sequestration [75]. Since botanical extract feeding decreases the accumulation of dP62 and ubiquitinated proteins in aged flies, we studied the ratio between ATG8-II and ATG8-I in flies fed with rosemary and ginger extracts. Western blot analysis shows that rosemary and rosemary plus ginger increases the ratio between ATG8-II and ATG8-I (Fig. 4G, and quantification in 4H-I). Together, these results indicate that feeding ethanol extracts of rosemary and ginger can improve proteostasis and activate autophagy during aging.

Given that AMPK is a key activator of autophagy [25], we set out to examine whether feeding rosemary and/or ginger extracts can increase AMPK activity. To do so, we measured phosphorylation of the catalytic subunit of AMPK at Thr184. Western blot analysis using a phosphospecific antibody revealed a significant increase in phospho-Thr184-AMPK levels in flies fed the combination of rosemary plus ginger extracts compared to controls (Fig. 4L, quantification in 4M). Interestingly, we failed to observe an increase in AMPK activation in flies fed either rosemary or ginger extracts separately. This result indicates that the combination of rosemary and ginger is important for AMPK activation.

### Ethanol-water extraction of rosemary plus ginger improves *Drosophila* lifespan and healthspan

**Figure 5.**
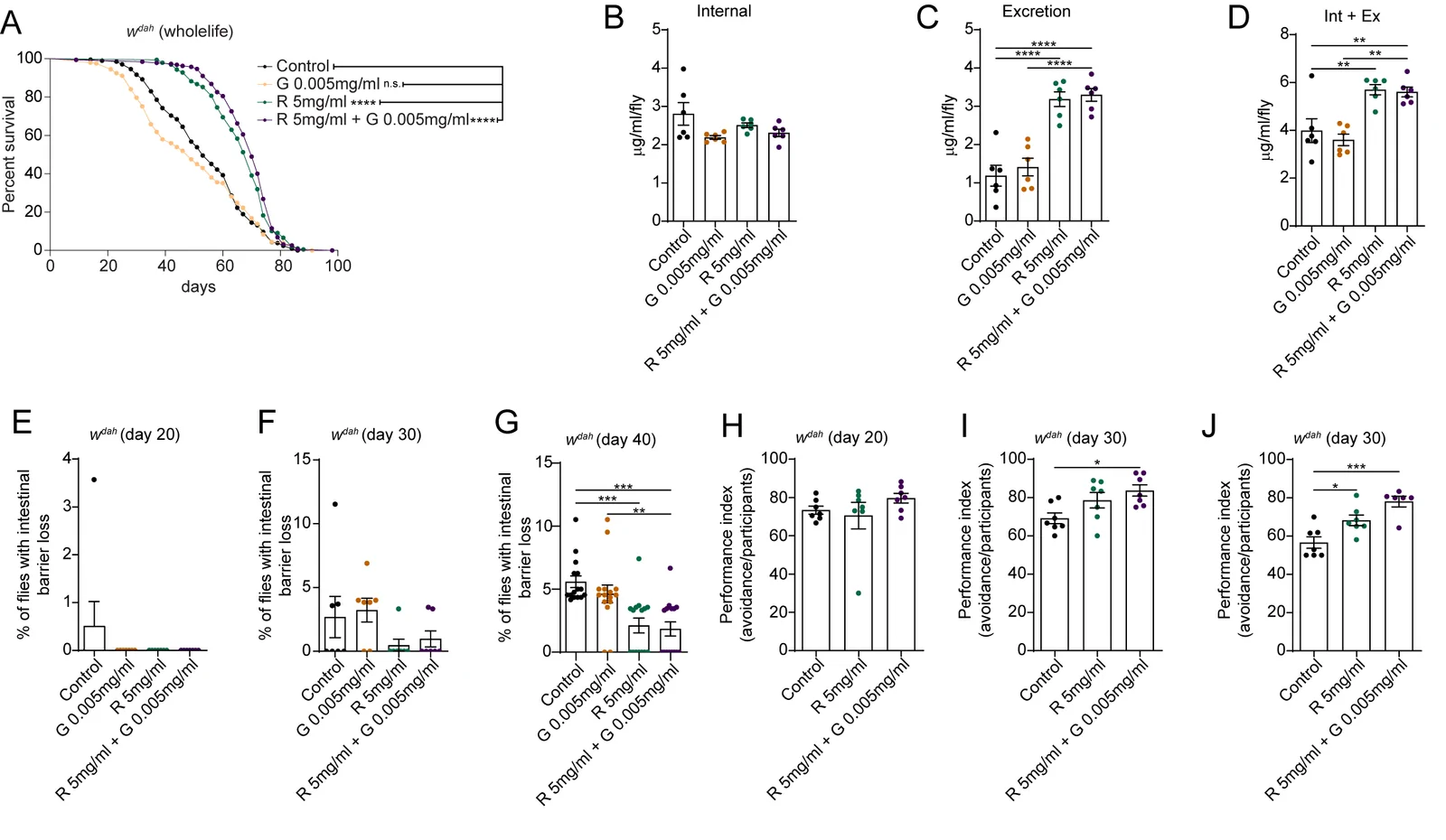
**Effects of Ethanol-water extraction of rosemary and ginger on *Drosophila* lifespan and healthspan**. (**A**) Survival curves of *w^dah^* female flies fed from day 3 onwards with ethanol (control), ginger, rosemary, and rosemary/ginger. log-rank test. For statistical analysis see Table 3. (**B-D**) Spectrophotometry analysis of consumed food at day 7 of *w^dah^* female flies fed with ethanol (control), ginger, rosemary, and rosemary/ginger from day 7 onwards. (**B**) internal food, (C) excreted food and (D) internal + excreted (Int + Ex). Control n = 6, Ginger n = 5, Rosemary n = 6, and R/G n = 6 replicates of 10 flies per replicate. **p ≤ 0.01; ****p≤0.0001; Kruskall-Wallis/Dunn’s multiple-comparisons test. (**E-G**) Intestinal barrier loss in *w^dah^* female flies fed with ethanol (control), ginger, rosemary, and rosemary/ginger from day 3 onwards at day 20(E), 30(F), and 40(G). day 20 Control n = 7, Ginger n = 7, Rosemary n = 7, and R/G n = 7 vials with 30 flies per vial. day 30 Control n = 7, Ginger n = 7, Rosemary n = 7, and R/G n = 7 vials with 30 flies per vial. day 40 Control n = 15, Ginger n = 15, Rosemary n = 15, and R/G n = 15 vials with 30 flies per vial; * p ≤ 0.05; **p ≤ 0.01; ***p≤0.001; one-way ANOVA/Šídák multiple-comparisons test. (**H-J**) Performance index of olfactory aversion in *w^dah^* female flies at day 20(H), 30(I), and 40(J) fed from day 3 onwards with ethanol (control), ginger, rosemary, and rosemary/ginger. n6 replicates with 30 flies per replicates. * p ≤ 0.05; ***p≤0.001; one-way ANOVA/Šídák multiple-comparisons test.

Ethanol-water mixtures have been proposed as suitable solvents for botanical extraction because of the different polarity of both solvents and their acceptability for human consumption [76]. We next tested extracts of rosemary and ginger prepared via water-ethanol binary solvent extraction. Feeding rosemary extract or rosemary plus ginger extracts prepared via water-ethanol extraction prolongs *Drosophila* lifespan, while ginger extract alone reduces *Drosophila* lifespan (Fig. 5A and Supplementary Fig. 2A and B, Table 3).

**Table 3:** Ethanol-water extracts of Rosemary plus Ginger extend *Drosophila* lifespan.

Rosemary concentration (mg/ml)	Ginger concentration (mg/ml)	Median lifespan	p-value versus control	% median lifespan increase	n (flies)	
**0**	0	53			206	Replicate 1
**0**	0.005	49	0.0162	-7.5	202	
**5**	0	70	<0.0001	32.1	197	
**5**	0.005	70	<0.0001	32.1	190	
**0**	0	49			234	Replicate 2
**0**	0.005	53	0.0523	8.2	206	
**5**	0	60	<0.0001	22.4	201	
**5**	0.005	65	<0.0001	32.7	202	
**0**	0	51			177	Replicate 3
**0**	0.005	53	0.2984	3.9	177	
**5**	0	64	<0.0001	25.5	174	
**5**	0.005	67	<0.0001	31.4	165	
**0**	0	49			179	
**0.1**	0	49	0.5991	0	132	
**0.5**	0	49	0.3504	0	150	
**2.5**	0	53	0.0121	8.2	149	
**5**	0	53	0.0009	8.2	150	
**0**	0	53			238	
**0**	0.005	42	0.0119	-20.8	233	
**0**	0.01	42	0.0087	-20.8	220	
**0**	0.05	42	0.0018	-20.8	213	

To validate that any longevity promoting effects were not due to reduced food intake, we analyzed *Drosophila* consumption of solid food by Con-Ex assay after seven days of extract feeding. Feeding these extracts of rosemary, ginger and rosemary plus ginger for one week to *w^dah^* flies does not affect the amount of internal food. However, rosemary and rosemary plus ginger extracts feeding increased the amount of excreted food affecting the overall food intake (Fig. 5B-D). Therefore, feeding these extracts of rosemary as well as rosemary plus ginger actually increases overall food intake and may decrease gut transit time. Next, we examined whether these extracts of rosemary and/or ginger can counteract intestinal aging. Using the ‘Smurf assay’, we analyze intestinal barrier integrity at days 20, 30, and 40 flies fed with rosemary, ginger, and rosemary plus ginger since day 3 (Fig. 5E-G). There was no significant reduction in intestinal barrier dysfunction, upon extract feeding, after 20 or 30 days (Fig. 5E and F). However, feeding rosemary alone or rosemary plus ginger for 40 days to *w^dah^* flies produced a significant reduction in the number of flies with intestinal barrier integrity loss (Fig. 5G). These results show that the prolongevity effects of feeding these extracts are linked to improved intestinal barrier function during aging.

Feeding the ethanol-water extract of ginger alone did not prolong lifespan or counteract age-onset intestinal barrier dysfunction. Hence, for the remainder of the study, we focused upon rosemary extract and the combination of rosemary plus ginger extract. To assess physiological brain function during aging, we tested associative learning and memory using olfaction aversion training [63]. Briefly, flies were conditioned to associate a neutral odor (3-octanol, OCT) with a series of electric shocks. After one hour of rest, they were placed in a T-maze and allowed to choose between OCT and a second neutral odor (4-methylcyclohexanol). Young control flies avoided the shock-associated OCT significantly more than aged flies (Fig. 5H-J; Supp. Fig. 2C). Furthermore, aged flies that had been fed both rosemary and ginger extracts showed a significantly better memory recall response than age-matched controls at day 30 (Fig. 5I). Aged flies that had been fed rosemary alone or rosemary plus ginger for 40 days also significantly improved associative learning and memory (Fig. 5J). Remarkably, the impact of feeding both rosemary and ginger was more pronounced than feeding rosemary alone at both the day 30 and day 40 timepoints. Hence, feeding a combination of rosemary and ginger extracts improved learning and memory in aged flies.

## DISCUSSION

In this study, we examined the impact of a number of different chloroform botanical extracts on fly lifespan. Of the extracts tested, rosemary or rosemary plus ginger showed prolongevity effects. Critically, we showed that the prolongevity effects were not linked to reduced feeding behavior. We then demonstrated that feeding these extracts of rosemary and ginger can counteract age-onset intestinal barrier dysfunction and improve proteostasis in aged flies. We validated and expanded upon these findings using ethanol extracts of rosemary and ginger. More specifically, we showed that ethanol extracts of rosemary and ginger also prolong lifespan and counteract age-onset intestinal barrier dysfunction. With these ethanol extracts of rosemary and ginger, we assayed both consumption and excretion to rule out the possibility that reduced feeding behavior was a confounding variable. In fact, we found that the anti-aging effects of ethanol extracts of ginger or rosemary plus ginger was linked to an overall increase in food intake. Interestingly, we found that feeding ethanol extracts of rosemary plus ginger improved markers of proteostasis, whereas the individual extracts did not. Moreover, we found that the combination of rosemary plus ginger extracts lead to increased AMPK activation. Finally, we sought to validate whether water-ethanol extracts of rosemary, ginger, or rosemary plus ginger could prolong lifespan and/or healthspan. Interestingly, we failed to observe prolongevity effects with a water-ethanol extract of ginger. However, the water-ethanol extract of rosemary alone and also the combination of water-ethanol extracts of rosemary plus ginger were both able to prolong lifespan and counteract age-onset intestinal barrier dysfunction. Critically, we also found that the combination of these extracts of rosemary plus ginger improved cognitive function in aged flies. Indeed, the impact of this combination of water-ethanol extracts of rosemary and ginger produced a more pronounced improvement in cognition than rosemary extract alone. Together, our findings support the idea that rosemary and ginger extracts can prolong healthspan in flies. It is worth noting that in our study we tested a slightly higher concentration of Rosemary extract than in a prior study using a high fat diet [59].

We have linked the observed healthspan-promoting effects with these botanical extracts to various cellular hallmarks of aging. Notably, we find that feeding flies extracts of rosemary and ginger improves markers of autophagy and proteostasis in aged flies. Although we do not know the cellular mechanism by which these botanical extracts promote autophagy, it is noteworthy that the combination of Ethanol extraction of rosemary plus ginger leads to increased AMPK activity. One limitation of this study, however, is that we have not examined whether either AMPK or autophagy is required for the healthspan-promoting effects of the extracts. Studies in both flies and mice have shown that maintaining intestinal barrier function during aging is sufficient to promote longevity [7]. Hence, it is plausible that the impact of rosemary and ginger extracts on systemic health is mediated via improvements in intestinal homeostasis. In addition, it is noteworthy that the longevity-promoting effects were associated with increased excretion of food. One interpretation of this finding is that the interventions that promote healthspan reduce gut transit time. It is, therefore, interesting to speculate that reduced gut transit time may play a role in promoting healthy aging in response to these botanical extracts. Indeed, constipation and altered bowel habits have been linked to Parkinson’s disease [77], Alzheimer’s disease [78], and a higher risk of ischemic heart disease and chronic kidney disease [79]. In the case of at least PD, altered gut transit time often precedes the onset of symptoms by years [77]. Of course, the relationships between gut transit time, intestinal health and systemic health are complicated and the causal relationships are not clear at the moment.

For investigation and applications related to botanical extracts, research design may be complicated by the chemical complexity and inherent variability of the extracts [80]. It is interesting that while we observe that three different extraction methods produced rosemary extracts with prolongevity effect, this was not the case for ginger extracts. We failed to observe significant prolongevity effects with water-ethanol or chloroform extracts of ginger. Remarkably, however, when water-ethanol extracts of both rosemary and ginger were combined they produced improvements in cognitive function which were more pronounced than rosemary alone. An additional limitation of this study is that we have not identified the active components within the extracts that confer improvements in heathspan. It will be interesting to determine if individual components can recapitulate the beneficial effects of individual extracts or of combinations of both extracts. In any case, our findings support the idea that botanical extracts of rosemary and ginger can be utilized to promote healthspan.

## Supplementary Materials

The Supplementary data can be found online at: www.aginganddisease.org/EN/10.14336/AD.2024.1558.
