# Supplementary material for: The Impact of Rosemary and Ginger Extracts on Aging and Healthspan in Drosophila
Source: Aging Dis. 2025 Feb 26;17(1):416–31. doi: 10.14336/AD.2024.1558 (PMC12727075; doi:10.14336/AD.2024.1558)
Supplement: Supplementary file 1 — The Supplementary data can be found online at: www.aginganddisease.org/EN/10.14336/AD.2024.1558. [file AD-17-1-416-s.pdf]

SUPPLEMENTARY DATA

The Impact of Rosemary and Ginger Extracts on Aging and Healthspan in *Drosophila*

Ricardo Aparicio, Anna M. Salazar, Edward T. Schmid, Armen Khanbabaei, Arun Rajgopal, R. Keith Randolph, David W. Walker

© 2025. Aparicio R. et al. Published online at http://www.aginganddisease.org/EN/10.14336/AD.2024.1558

SUPPLEMENTARY DATA

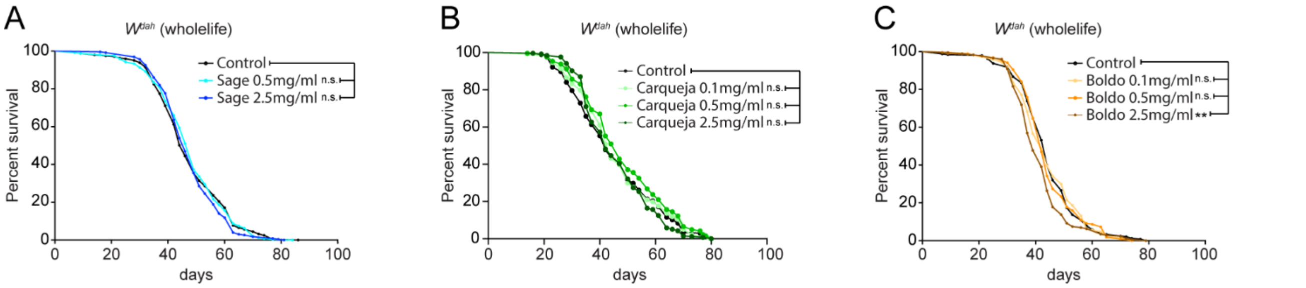

Supplementary Figure 1. Chloroform extraction of Sage, Carqueja, and Boldo does not extend *Drosophila* lifespan. (A-C) Survival curves of w^{dah} female flies fed from day 3 onwards with different botanical extracts at different concentrations. (A) sage, (B) carqueja, and (C) boldo, log-rank test. For statistical analysis see Table 1.

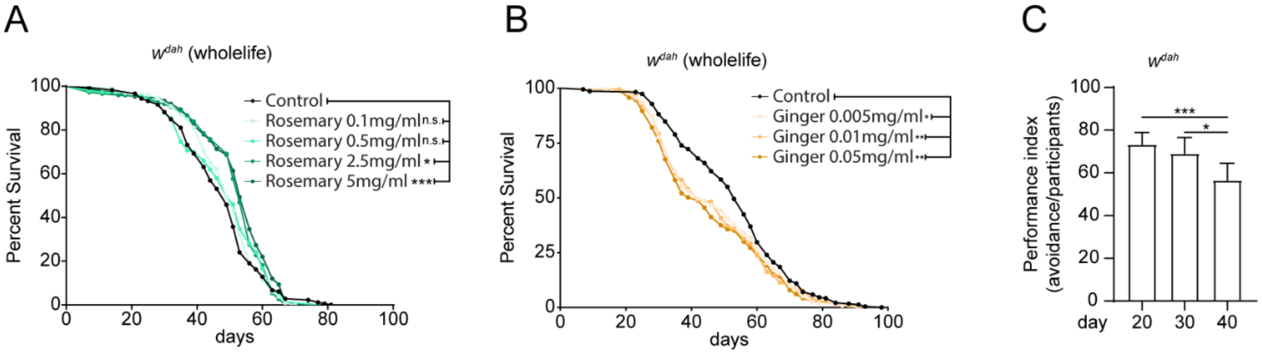

Supplementary Figure 2. Lifespan of Water-ethanol extraction of rosemary and Ginger in flies. (**A and B**) Survival curves of w^{dah} female flies fed from day 3 onwards with water-ethanol extraction of rosemary (A) and ginger (B) at different concentrations. Log-rank test. For statistical analysis see Table 3. (**C**) Performance index of olfactory aversion in control female flies (w^{dah}) at day 20, 30, and 40. n=6 replicates with 30 flies per replicates. * $p \leq 0.05$; *** $p \leq 0.001$; one-way ANOVA/Šídák's multiple comparisons test.

© 2025. Aparicio R. et al. Published online at http://www.aginganddisease.org/EN/10.14336/AD.2024.1558
